# Personalized Medicine for the Management of Non-Communicable Diseases (NCDs)

**DOI:** 10.3390/jcm15166261

**Published:** 2026-08-13

**Authors:** Alessandro Mattina, Giulio Geraci, Vincenzina Lo Re

**Affiliations:** 1 UPMC Italy, 90127 Palermo, Italy; vlore@ismett.edu; 2 Diabetes Service, IRCCS ISMETT, 90127 Palermo, Italy; 3 Department of Medicine and Surgery, “Kore” University of Enna, 94100 Enna, Italy; giulio.geraci@unikore.it; 4 Internal Medicine Unit, Hospital Umberto I, 94100 Enna, Italy; 5 Neurology Service, IRCCS ISMETT, 90127 Palermo, Italy

## 1. Introduction

Non-communicable diseases (NCDs) are the leading cause of death worldwide and are among the major drivers of healthcare expenditure, particularly in societies undergoing progressive population aging [1]. Their onset, progression to complications, and response to treatment are influenced by the interaction of multiple factors, especially genetic, metabolic, and environmental determinants, as well as by differences in access to high-quality care.

Advances in epidemiological research, clinical phenotyping, imaging, molecular biology, and digital data analysis have created new opportunities for more individualized prevention and treatment [2]. Each NCD may be more appropriately viewed as a heterogeneous condition encompassing distinct subgroups with different clinical trajectories and complication profiles, rather than as a single homogeneous disease.

The main challenge, therefore, is not simply to generate increasing amounts of data, but to determine which information is clinically relevant, for which patient, and at which stage of the disease course.

Important gaps remain. Most stratification tools have not been externally validated, adequately calibrated, or evaluated for their impact on clinical decision-making and patient outcomes [3]. Many candidate biomarkers fail to progress from clinical research to routine practice [4]. Risk scores are often developed in highly selected populations and subsequently applied to groups that were poorly represented in the derivation cohorts, including older adults, people with multimorbidity, frail individuals, and populations living in low- and middle-income countries [5]. Sex is still too often considered only in post hoc subgroup analyses rather than being prospectively incorporated into study design and analysis [6]. Moreover, personalization has focused mainly on diagnosis and drug selection, with less attention paid to long-term care pathways, transitions between care settings, and prognosis.

Against this background, this Editorial uses personalized medicine as a broad clinical concept encompassing both precision approaches based on detailed biological or multidimensional information and stratified approaches based on clinically meaningful risk profiles or phenotypes. The Special Issue includes ten original studies (Contributions 1–10) and two reviews (Contributions 11 and 12), addressing different components of the care continuum for NCDs, from population surveillance, prevention, and screening to prognostic stratification, biomarker evaluation, and phenotype-guided treatment. To provide a coherent framework across these heterogeneous contributions, we synthesize them within three complementary domains: population risk and prevention, evolving disease trajectories, and biomarkers and integrated clinical phenotypes.

## 2. From Population Risk to Targeted Prevention

The first step toward individualized care is an accurate understanding of how risk is distributed. Global forecasts indicate that annual diabetes-related mortality may reach approximately 1.63 million deaths by 2030, with type 2 diabetes accounting for most of the burden and the least favorable trends concentrated among younger and middle-aged adults, particularly in Southeast Asia and in low- and middle-income countries (Contribution 1). National data from Sri Lanka provide a complementary perspective (Contribution 2). Over two decades, total mortality attributed to diabetes increased by 169%, whereas the corresponding increase in hospital mortality was substantially smaller. The divergence between deaths recorded overall and those occurring in hospitals suggests that disease burden cannot be interpreted independently of access to diagnosis, continuity of care, and the capacity of healthcare systems to manage chronic risk outside acute-care settings. This interpretation is consistent with persistent global gaps in diabetes diagnosis and treatment [7].

These findings highlight the need not only to improve the management of established disease, but also to intervene earlier, before modifiable risk factors become firmly established [8]. In this context, Alqahtani et al. examined school-aged children and adolescents in the Aseer Region of Saudi Arabia and confirmed that, within their regional setting, overweight and obesity were associated with both family history and modifiable behaviors, including frequent fast-food consumption, low fruit and vegetable intake, physical inactivity, prolonged screen time, and short sleep duration (Contribution 3).

Alongside preventive strategies, screening is particularly important for conditions such as diabetes, which remain substantially underdiagnosed and often come to clinical attention only after complications have developed [7]. In this context, Bashkin et al. evaluated postprandial glucose screening in hospital visitors without known diabetes (Contribution 4). They identified postprandial hyperglycemia in 7.8% of participants in the propensity-matched cohort, with prevalence ranging from 6.0% to 9.4% across the groups examined. Age above 50 years was the most consistently associated factor, whereas associations with obesity, self-reported health status, and vegetable intake differed between groups. Ethnocultural categories should not, however, be interpreted as biological explanations, but may instead reflect differences in dietary patterns, socioeconomic conditions, and access to healthcare. Overall, these findings support the feasibility of opportunistic postprandial glucose assessment in this specific hospital-based setting, but do not establish the effectiveness or clinical utility of population-level screening.

Taken together, these studies illustrate how population surveillance, prevention, and targeted screening can contribute to identifying groups in whom earlier assessment may be warranted, thereby supporting more targeted preventive strategies.

## 3. Personalizing Care Across Disease Trajectories

Once an NCD has developed, risk remains dynamic. A five-year study including all older, multimorbid patients discharged from an Internal Medicine ward showed a post-discharge mortality rate of 57%, with a marked early peak of 32% during the first year (Contribution 5). Importantly, the predictors of mortality differed across the early, intermediate, and late phases of follow-up. A single-risk estimate at discharge is therefore insufficient. Personalized follow-up should include periodic reassessment of risk, allowing the intensity and goals of care to be adjusted as the patient’s clinical condition evolves.

This temporal perspective also influences how treatment success should be defined. In patients with severe multimorbidity or frailty [5], intensifying disease-specific treatment may not always represent the most appropriate objective. In this setting, personalization concerns not only which intervention should be offered, but also whether and when its expected benefit remains aligned with the patient’s overall prognosis.

Clinical heterogeneity may also be reflected in symptoms that are overlooked when attention is focused on the primary diagnosis. Exocrine pancreatic insufficiency is relevant in diabetes because reduced exocrine function may occur in both type 1 and type 2 diabetes. However, gastrointestinal manifestations may be incorrectly attributed to other causes. In an online survey, gastrointestinal symptom burden was high among participants with exocrine pancreatic insufficiency (EPI), regardless of diabetes status, and the EPI Symptom Score was able to distinguish probable exocrine insufficiency from other gastrointestinal conditions (Contribution 6).

These studies show that individualized care across the disease course requires both repeated reassessment of prognosis and attention to clinically relevant manifestations that may not be adequately captured by the primary diagnosis alone.

## 4. From Risk Markers to Integrated Clinical Phenotypes

Biomarkers and intermediate phenotypes may improve risk stratification when they capture aspects that are not represented by conventional variables [4]. Familial hypercholesterolemia provides a clear example, as patients with the same genetic diagnosis and apparently similar lipid profiles may exhibit substantially different degrees of vascular damage. In 100 genetically characterized patients with heterozygous familial hypercholesterolemia, coronary artery calcium was associated with both pulse wave velocity and cumulative low-density lipoprotein cholesterol burden, and its association with arterial stiffness persisted after adjustment for conventional risk factors and treatment (Contribution 7). Integrating lifetime lipid exposure, vascular function, and coronary imaging may therefore help distinguish patients who require different intensities or timing of preventive treatment despite belonging to the same diagnostic category.

At a more exploratory level, microbiome analysis illustrates both the potential and the limitations of detailed biological phenotyping. In an older population with type 2 diabetes, biological sex did not significantly influence overall gut microbiota diversity. However, several exploratory associations between specific microbial taxa and metabolic parameters differed between men and women (Contribution 8). These hypothesis-generating findings support further investigation of sex- and age-related factors in microbiome studies in type 2 diabetes, but require independent validation before clinical implications can be established.

Within the broader context of precision medicine in metabolic disease, two related laboratory studies assessed whether routine full blood count parameters could provide additional information on glycated hemoglobin and estimated whole-blood viscosity in patients with diabetes. The first found that red blood cell variables showed more consistent relationships with changes in glycemic control and estimated viscosity than platelet- or lymphocyte-derived ratios (Contribution 9). The subsequent exploratory analysis used Akaike Information Criterion-based regression to identify full blood count parameters retained in models of glycated hemoglobin and estimated whole-blood viscosity (Contribution 10). Red cell distribution width, mean corpuscular volume, red blood cell count, and other routine parameters emerged as potential contributors, although the analysis did not develop or validate a clinical prediction model. These findings should therefore be regarded as exploratory associations until their potential predictive value has been assessed and independently validated.

The first of the two reviews included in this Special Issue extends this selective, clinically oriented approach to thrombophilia evaluation. Genetic variants provide stable information on inherited susceptibility, whereas circulating biomarkers reflect dynamic processes involving coagulation, inflammation, and endothelial activation, but are more susceptible to multiple confounding factors (Contribution 11). Emerging biomarkers may improve thrombotic risk assessment, but indiscriminate testing can produce ambiguous findings and promote over-testing and potentially inappropriate anticoagulation. A personalized strategy should therefore begin with a clearly defined clinical question and use testing only when the result has the potential to influence management [9].

The second review focuses on integrated clinical phenotyping in type 2 diabetes, in which cardiovascular risk cannot be explained by hyperglycemia alone. Atherosclerotic cardiovascular disease, heart failure, chronic kidney disease, obesity, metabolic liver disease, dyslipidemia, hypertension, and microvascular dysfunction may coexist with different relative contributions (Contribution 12). Identifying the predominant phenotype allows therapeutic priorities to be aligned with organ-specific risk, using glucose-lowering therapies with cardiovascular benefit alongside other individualized strategies. The phenotype should also be reassessed over time, as treatment response and disease progression may alter the balance of risk.

Collectively, these contributions show how biomarkers and multidimensional clinical phenotyping may refine risk stratification, provided that their added value is validated and translated into meaningful changes in clinical management.

## 5. Conclusions

Several common limitations emerge across these domains. Many findings are derived from cross-sectional, retrospective, single-center, or explicitly exploratory analyses. Small sample sizes and geographic concentration limit generalizability, while proposed symptom assessment tools and biomarkers often lack external validation. Longitudinal changes are frequently inferred from isolated measurements, and underrepresented populations, including frail patients and those living in resource-limited settings, remain insufficiently represented in derivation cohorts. Prospective, multicenter studies should therefore include adequate demographic and clinical diversity, together with sufficiently long follow-ups, to capture changes in phenotype and treatment response.

The crucial question is not whether a new marker is statistically significant, but whether its use changes clinical management, improves patient outcomes, and does so without disproportionate cost or complexity. Advanced molecular and imaging tools may be appropriate for selected high-risk patients, but personalized medicine cannot rely exclusively on resource-intensive technologies. The integration of routine laboratory data, structured symptom assessment, longitudinal follow-up, and traditional clinical evaluation may provide scalable alternatives. These strategies should be held to the same standards of validation and clinical utility as novel biomarkers, together with the formal assessment of cost-effectiveness and interpretability. Their implementation also requires interoperable data systems and sustainable integration into clinical workflows, with attention to clinician workload, algorithmic bias, data privacy, and patient preferences. Unequal access to advanced diagnostic technologies and digital infrastructure must also be considered, particularly across healthcare systems with substantially different resources.

Progress in personalized medicine research will depend not only on identifying new variables, but, more importantly, on integrating them into clinical decision-making so that each patient receives the right intervention, at the right intensity, and at the most appropriate point in their care pathway.

## Data Availability

No new data were created or analyzed in this study. Data sharing is not applicable to this article.
